# Metabolic consequences of erastin-induced ferroptosis in human ovarian cancer cells: an untargeted metabolomics study

**DOI:** 10.3389/fmolb.2024.1520876

**Published:** 2025-01-20

**Authors:** Kaylie I. Kirkwood-Donelson, Alan K. Jarmusch, Carl D. Bortner, Bruce Alex Merrick, Birandra K. Sinha

**Affiliations:** ^1^ Metabolomics Core Facility, Immunity, Inflammation, and Disease Laboratory, National Institute of Environmental Health Sciences, National Institutes of Health, Research Triangle Park, NC, United States; ^2^ Laboratory of Signal Transduction, Research Triangle Park, NC, United States; ^3^ Mechanistic Toxicology Branch, Division of Translational Toxicology, National Institutes of Environmental Health, NIH, Research Triangle Park, NC, United States

**Keywords:** ferroptosis, biomarkers, ovarian cancer, erastin, oxidative stress

## Abstract

**Introduction:**

Ovarian cancer has been difficult to cure due to acquired or intrinsic resistance and therefore, newer or more effective drugs/approaches are needed for a successful treatment in the clinic. Erastin (ER), a ferroptosis inducer, kills tumor cells by generating and accumulating reactive oxygen species (ROS) within the cell, resulting in an iron-dependent oxidative damage-mediated ferroptotic cell death.

**Methods:**

We have utilized human ovarian cancer cell lines, OVCAR-8 and its adriamycin-selected, multi-drug resistance protein (MDR1)-expressing NCI/ADR-RES, both equally sensitive to ER, to identify metabolic biomarkers of ferroptosis.

**Results:**

Our studies showed that ER treatment rapidly depleted cellular glutathione and cysteine and enhanced formation of ophthalamate (OPH) in both cells. Opthalalmate has been proposed to be a biomarker of oxidative stress in cells. Our study also found significant decreases in cellular taurine, a natural antioxidant in cells. Additionally, we found that ER treatment decreased cellular levels of NAD+/NADP+, carnitines and glutamine/glutamate in both cells, suggesting significant oxidative stress, decrease in energy production, and cellular and mitochondrial disfunctions, leading to cell death.

**Conclusion:**

Our studies identified several potential biomarkers of ER-induced ferroptosis including OPH, taurine, NAD+, NADP+ and glutamate in ovarian cancer cells. Identifying specific metabolic biomarkers that are predictive of whether a cancer is susceptible to ferroptosis will help us devise more successful treatment modalities.

## Introduction

Ovarian cancer is one of the most common and lethal malignancy of the female reproductive system (Cortez et al., 2018; Wang et al., 2024). Almost 70%–75% of patients are diagnosed at an advanced stage (stage III and IV) with distant metastasis. Many patients receiving standard therapy (cytoreductive surgery followed by adjuvant chemotherapy) develop chemotherapy resistance, resulting in a poor survival rate of approximately 30%–40% worldwide (Wang et al., 2024). Although use of poly (adenosine diphosphate-ribose) polymerase (PARP) inhibitors has increased progression-free survival, unfortunately, many patients do not respond to PARPi treatment due to intrinsic or acquired resistance. Drug resistance is now a major challenge in the treatment of ovarian cancers and is the primary contributor to poor prognosis.

Recently, inducers that cause an iron-mediated mode of cell death known as ferroptosis (e.g., Erastin and its related compounds) have become a promising treatment option for cancer therapy, including cancers that are resistant to standard therapy. Ferroptosis involves the accumulation of intracellular lipid hydroperoxides (LOOH) due to the formation of reactive oxygen species (ROS) and inhibition of glutathione peroxidase 4 (GPX4), resulting in the damage to cellular membranes (lipid peroxidation) in the presence of iron (Dixon et al., 2012; Dixon et al., 2014). The damaging species is the reactive hydroxyl radical (^●^OH), formed from the reaction of H_2_O_2_ with Fe^2+^ (the Fenton reaction). Erastin (ER) induces cell death in various tumor cells by ferroptosis that involves disrupting the cellular antioxidant defense system and triggering lipid peroxidation (Galaris et al., 2019; Yang and Stockwell, 2016). However, other mechanisms of ER-dependent tumor cell death have also been proposed, including the inhibition of X_C_^–^ (glutamate/cystine antiporter) system, the inhibition of the mitochondria-bound voltage-dependent anion channel (VDAC) and the modulation of the tumor suppressor p53 gene (Zhao et al., 2020; Yagoda et al., 2007; Jiang et al., 2015; Kang et al., 2019). Inhibition of system Xc-by ER leads to a decrease in intracellular cystine, a precursor for the synthesis of glutathione (GSH). This decrease in cellular GSH results in an increase in ROS formation (and oxidative stress), resulting in cellular damage and death (Sui et al., 2018). ER also inhibits VDAC functions which plays an important role in the induction of ferroptosis (Maldonado et al., 2013). VDAC, an ion channel located in the outer mitochondrial membrane, mediates and controls molecular and ion exchange between the mitochondria and the cytoplasm. The permeability of the VDAC has been shown to be altered by certain drugs, resulting in mitochondrial dysfunction, ROS production, and cell death (Yagoda et al., 2007; DeHart et al., 2018).

ER and its analogs can also sensitize resistant tumor cells to chemotherapy agents (Yu et al., 2015; Cheng et al., 2021) thus, understanding the mechanisms of ER cytotoxicity becomes crucial for developing strategies to design better drug combinations for the treatment of human cancers. Drug development in cancer has been difficult and problematic as most drugs suffer from a high rate of failure in the clinic with approximately 9 of 10 drugs failing to reach the market. To improve the success rate and the effectiveness of cancer drug development, additional biomarkers that are more informative and can predicate success (or failure) earlier in pre-clinical settings are needed (Kuang et al., 2023).

Over the past several years, research focused on identifying and investigating various biomarkers for cancer has increased (Kuang et al., 2023; Mendez Hernandez and Ramasco Rueda, 2023), leading to significant advancements in the screening, diagnosis, and treatment of human cancers in clinical settings. Biomarkers are integral to cancer drug development as the effects of chemotherapeutics must be evaluated during the clinical trials. Thus, it is extremely important to have an extensive catalog of biomarkers that can be utilized to evaluate various effects of investigational drugs in humans.

Unfortunately, biomarkers of ferroptosis are similar to other types of regulated cell death or pathological conditions (Chen et al., 2021). Therefore, a clear understanding of specific biomarkers and contributors of ferroptosis would provide new opportunities for developing treatments for cancers and other diseases involving iron overload. Furthermore, a combination of multiple biomarkers may also help detect ferroptotic cell death in the clinic. However, the challenge remains how to utilize basic research findings into clinical applications. Solving these challenges requires clear understanding of the molecular mechanisms and signal transduction of ferroptosis, as well as the use of new technologies, such as metabolomics, to discover specific biomarkers. Recent advances in metabolomic research has allowed altered metabolism in cancer to be novel source for detecting new biomarkers for cancer diagnosis, prognosis and treatment success (Chen et al., 2021).

### Materials and methods

Erastin, (2-[1-[4-[2-(4-Chlorophenoxy) acetyl]-1-piperazinyl]ethyl]-3-(2-ethoxyphenyl)-4(3H)-quinazolinone) was obtained from Cayman Chemicals (Ann Arbor, MI) and was dissolved in DMSO. Stock solutions were stored at −80C. Fresh drug solutions, prepared from the stock solutions, were used in all experiments.

### Cell culture

Authenticated human ovarian OVCAR-8 and ADR-selected OVCAR-8 cells (Batist et al., 1986; Cowan et al., 1986) (NCI/ADR-RES cells) were obtained from the Division of Cancer Treatment and Diagnosis Tumor Repository, National Cancer Institute the NCI-Frederick Cancer Center (Frederick, MD, United States). The NCI/ADR-RES cells express MDR1 and have higher activities of SOD, Catalase, and GSH-dependent peroxidase1, and transferase (Batist et al., 1986; Cowan et al., 1986). However, ER was similarly cytotoxic to both cells (IC_50_ 1.2 ± 0.10 × 10^-6^ M vs. 0.8 ± 0.15 × 10^-6^ M, for OVCAR-8 and NCI/ADR-RES cells, respectively (Sinha et al., 2024)). Cells were grown in Phenol Red-free RPMI 1640 media (pH = 7.0) supplemented with 10% fetal bovine serum and antibiotics. Cell cultures were incubated in an incubator at 37°C and 5% CO2 with saturating humidity. Cells were routinely used for 20–25 passages, after which the cells were discarded, and a new cell culture was started from the frozen stock.

### Metabolomics Sample collection and preparation

Cells were treated with ER (2.5 µM) for 24 h, washed once with ice-cold PBS (pH 7.4) and collected in methanol by scraping. Dried cell lysates were stored at −80°C until analysis and then resuspended in 80 µL of 98:2 water/acetonitrile. System blanks comprised of the same resuspension solvent and a pooled quality control (QC) sample comprised of 15 µL aliquots of each sample were generated.

### Metabolomics instrumentation

Samples were analyzed on a Vanquish Horizon ultra-high performance liquid chromatography system (UPLC, Thermo Scientific) coupled to an Orbitrap Lumos Tribrid high-resolution mass spectrometer (HRMS, Thermo Scientific). An EASY-Max NG ionization source was operated in the heated-electrospray ionization configuration. The source parameters were as follows: spray voltage of +4,000 V in positive ionization mode or −3,000 V in negative ionization mode, sheath gas of 50 arbitrary units (arb), auxiliary gas of 10 arb, sweep gas of 1 arb, ion transfer tube at 325°C, vaporizer at 350°C. Prior to measurement, the mass spectrometer was calibrated using FlexMix (Thermo Scientific). EASY-IC (Thermo Scientific) fluoranthene was used during data collection as a lock mass.

Chromatographic separation was carried out on a Kinetex F5 analytical column (2.1 inner diameter, 100 mm length, 100 Å, 2.6 µm particle size, Phenomenex) with corresponding guard cartridge at 30°C. Gradient elution was performed using HPLC-grade water with 0.1% acetic acid (A) and HPLC-grade acetonitrile with 0.1% acetic acid (B). Separation was performed as follows: 0% B from 0–2.0 min, 0%–100% B from 2.0 to 10.5 min, 100% B from 10.5 to 12.0 min, 100%–0% B from 12.0 to 13.0 min, 0% B from 13.0 to 20.0 min at a flow rate of 500 μL/min.

### Metabolomics data acquisition

Prior to acquiring liquid chromatography-mass spectrometry (LC-MS) sample data, liquid chromatography-tandem mass spectrometry (LC-MS/MS) data were acquired using the AcquireX deep scan methodology. For AcquireX acquisition, LC-MS data were collected for a system blank (exclusion list) and pooled QC (inclusion list), then LC-MS/MS data were collected over seven QC injections. MS data were acquired at 120,000 resolution from *m/z* 100-1,000 with an RF lens of 60% and maximum injection time of 50 m. MS/MS data were acquired at 30,000 resolution using an isolation width of *m/z* 1.5, stepped assisted higher-energy collision induced dissociation was used with energy steps of 20, 35, and 60 normalized collision energy, and a maximum injection time of 54 ms. An intensity filter was applied with an intensity threshold of 2E4. Dynamic exclusion was used with the following parameters: exclude after n = 3 times; if occurs within 15 s; exclusion duration of 6 s; a low mass tolerance of 5 ppm mass error; a high mass tolerance of 5 ppm mass error; and excluding isotopes.

LC-MS sample data were collected in positive and negative ionization mode from individual samples, system blanks, and pooled QCs. Samples and system blanks were injected at 4 µL. The pooled QC was injected at different volumes (triplicate injections at 2 μL, 4 μL, and 6 µL) for signal response evaluation (Overdahl et al., 2023). MS data were acquired at 120,000 resolution from *m/z* 100-1,000 with an RF lens of 60% and maximum injection time of 50 ms.

### Metabolomics data processing and annotation

Data files (.raw) were processed with Compound Discoverer 3.3.3.220 (ThermoFisher Scientific) to identify molecular features and, where possible, annotate them with chemical names. Features were annotated in CompoundDiscoverer based on MS/MS spectral matching in alignment with the Metabolomics Standards Initiative (Fiehn et al., 2007). Public, commercial and in-house MS/MS spectral libraries including NIST 2020, GNPS (access 04-01-2022), mzCloud (offline, endogenous metabolites) were utilized. An output table containing feature descriptors (*e.g., m/z* and retention time), annotation information, and peak areas were further processed using in-house R code via Jupyter Notebook for data formatting and analysis. Initial processing steps included formatting the output table from Compound Discoverer and assignment of MSI levels of confidence. MSI Level 2 features have an MS/MS match to either a database entry or an in-house library entry. MSI Level 4 and Level 5 features have either an MS/MS match but no database match, or no MS/MS match. Some MSI Level 2 features were manually promoted to MSI Level 1 after matching in-house *m/z* and retention time data acquired on the same analytical platform using identical conditions.

Subsequent processing steps included outlier removal, feature quality filtering via assessment of pooled QC signal response and dispersion ratio (i.e., comparison of signal variance in pooled QC replicate injections versus samples), data normalization, and multi- and univariate statistics. Sample outliers were identified and removed based on their total signal (i.e., sum of peak area) being greater than 1.5x the interquartile range. The signal response evaluation approach utilized here is detailed in Overdahl et al. (Overdahl et al., 2023) In short, data for each feature collected from the pooled QCs at three volumes was evaluated for a corresponding increase in signal. In this case, a Pearson correlation with *p* = 0.05 was used as the cutoff for significance, therefore only features displaying positive correlations within the *p* = 0.05 parameter were retained for further analysis. Feature abundances were row sum normalized to the total signal of all features for a given sample to account for differences in input material.

### Statistical analysis of metabolomics data

Statistical analyses were also performed using R via Jupyter Notebook. Data were first examined using unsupervised multivariate statistics via principal component analysis (PCA). Data were first log_10_-transformed to reduce the weighting of abundant features in the PCA computation. PCA score plots (PC1– PC3) and their corresponding loading plots were generated for both positive and negative mode data. Mid-level data fusion was utilized to combine the principal components obtained from PCA of positive and negative mode ionization mode data to give a singular visual output based on comprehensive metabolomics information. PCA plots were annotated according to genotype and treatment group in order to examine variance between sample groups as well as sample variance among those within a single group. Two-way ANOVA was used to detect significant differences followed by a post-hoc Tukey’s Honest Significance Difference (HSD) test for identifying pairwise differences while adjusting for multiple hypothesis testing. Log_2_ fold changes (FC) were also calculated for each pairwise comparisons. Features with an adjusted p-value <0.05 and a log_2_FC > 1 or < -1 were considered significantly statistically different. Volcano plots were generated for each comparison as well as box plots of peak areas for all statistically significant features.

### Determination of erastin-induced depolarization of membranes in ovarian cells

Determination of changes in plasma membrane potential was carried out using DiBAC4. Briefly, 30 min prior to time of examination, DiBAC_4_(3) (Invitrogen; Molecular Probes) was added to 1-mL of cells to a final concentration of 150 ng/mL and incubation was continued at 37°C, 7% CO_2_ atmosphere. Flow cytometric analysis was carried out using a BD Fortessa flow cytometer and FACSDiVA software (Becton Dickinson Immunocytometry Systems, San Jose, CA). Single cells were gated on a FCS-area versus width dot plot. DiBAC_4_(3) was excited at 488 nm and detected at 530 nm. For each sample, 10,000 cells were examined on a DiBAC_4_(3) histogram to determine the changes in mean fluorescent intensity (MFI). An increase or decrease in DiBAC_4_(3) fluorescence shows cellular depolarization or hyperpolarization, respectively.

## Results

### OVCAR-8 and NCI/ADR-RES cells have differential profiles and respond differentially to Erastin

**FIGURE 1:**
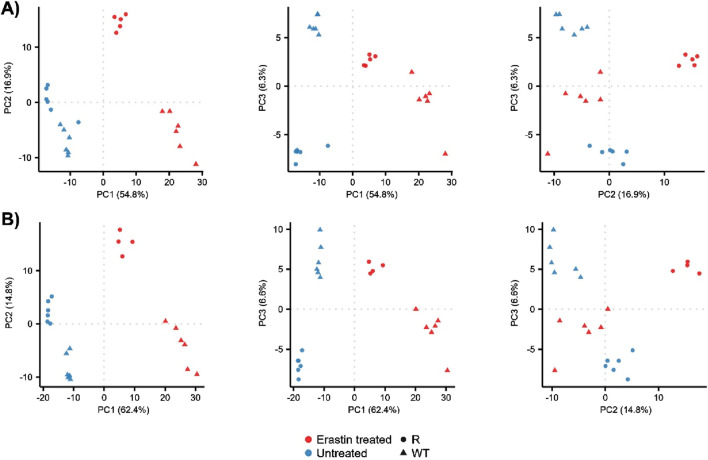
PCA of WT and R cells prior to and 24 h after Erastin treatment. PCA score plots for principal components 1 through 3: **(A)** positive ionization mode data and **(B)** negative ionization mode data.

The metabolomic profiles of OVCAR-8 (WT) and NCI/ADR-RES (R) cells were explored using principal component analysis (PCA), Figure 1 and Supplementary Figure S1. This approach allowed for the overall metabolomics profiles to be compared between samples using unsupervised multivariate statistics. Untreated (blue) WT and R groups were visually and statistically different in the positive ionization mode, Figure 1A, (PERMANOVA, F = 4.9, *p* = 0.005) as well as the negative ionization mode, Figure 1B, (F = 5.5, *p* = 0.006). Erastin treatment (red) resulted in the visual and statistical differentiation from untreated cells in the positive (F = 20, *p* = 0.001) and negative ionization mode (F = 24.2, *p* = 0.001) data as well as increased the visual separation between WT and R cells, implying differential response. Given the clear changes in metabolomic profiles between WT, R, and ER-treated cells, we investigated the individual metabolites which contributed to the observed profiles differences.

### Erastin induces changes in metabolomics redox

**FIGURE 2:**
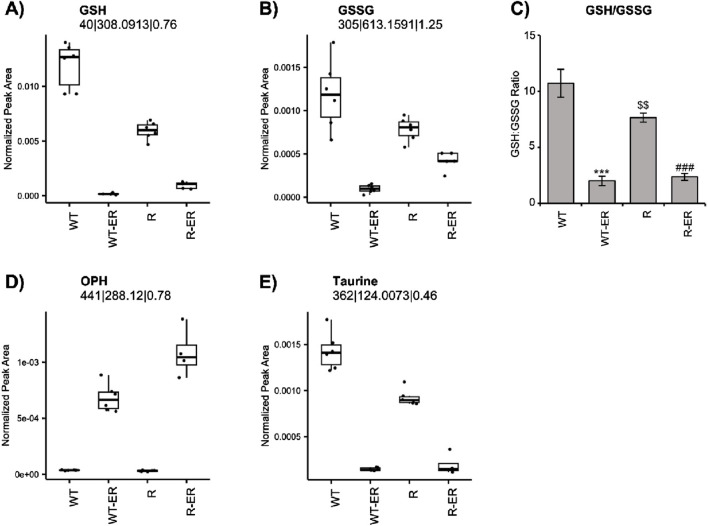
Redox changes resulting from Erastin (2.5 µM) treatment at 24 h in WT and R cells. Metabolomic measurement of **(A)** GSH **(B)** GSSG and **(C)** calculated levels of GSH/GSSG ratio. Error bars represent SD. Metabolomic measurement of levels of **(D)** OPH and **(E)** Taurine. ***, ### p values of <0.001 compared to untreated controls and $$ p values <0.005 compared to untreated WT, respectively.

Redox balance (i.e., balance of oxidants and antioxidants) is crucial in the cellular maintenance of reactive oxygen species (ROS). Both reduced form glutathione (GSH) and oxidized form glutathione disulfide (GSSG) are crucial cellular metabolites involved in redox balance. Metabolomics analysis indicated different levels of GSH and GSSG between WT and R cells (Figures 2A, B). Further, the ratio of GSH/GSSG, a metric of oxidative stress (Kemp et al., 2008; Chen et al., 2024) was found to be differential in the untreated condition (p-value = 0.03, fold change = 2.6) between WT and R cells (Figure 2C). Levels of GSH and GSSG as well as the GSH:GSSG ratio significantly decreased in WT and R cells in response to ER treatment.

Ophthalmic acid (OPH), formed via reaction of ɣ-glutamylcysteine and glutathione synthase, has been suggested to be a biomarker of oxidative stress in cells and tissues following depletion of cellular glutathione (Soga et al., 2006). Since ER treatment significantly decreased GSH in both OVCAR-8 and NCI/ADR-RES cells, we examined the formation of OPH in these cells. Untargeted metabolomics indicated a significant increase in OPH (Figure 2D) in ER-treated WT (p-value = 4.3 × 10^−8^, fold change = 18) and R (p-value = 1.0 × 10^−10^, fold change = 34) cells. Intriguingly, OPH levels were greater in ER-treated R cells.

Our metabolomic profile also indicated that ER treatment concomitantly decreased taurine formation in both cells. Taurine has been reported to be a natural antioxidant and biomarker of oxidative stress in cells (Surai et al., 2021; Baliou et al., 2021). Similar to GSH and GSSG, taurine was found to be significantly lower (p-value = 9.5 × 10^−6^) in untreated R cells compared to WT cells. Taurine levels significantly decreased in both WT and R cells in response to ER treatment (Figure 2E).

### Erastin decreases NAD+ and NADP+ in OVCAR-8 and NCI/ADR-RES cells

**FIGURE 3:**
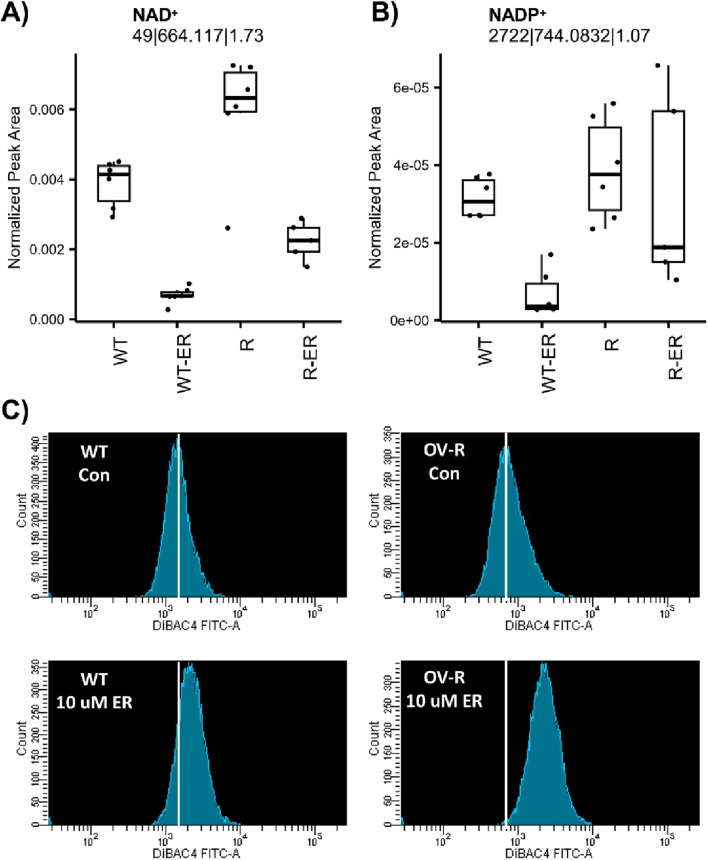
Effects of Erastin on metabolic-measured levels of NAD+ **(A)** and NADP+ **(B)** and plasma membrane depolarization **(C)** induced by Erastin (10 µM) in WT and R cells at 4 h treatment.

Nicotinamide adenine dinucleotide (NAD^+^) and nicotinamide adenine dinucleotide phosphate (NADP^+^) are essential for maintaining cellular redox homeostasis and for modulating numerous biological events, including cellular metabolism (Biniecka et al., 2023; McKay-Corkum et al., 2023). NAD^+^ was significantly depleted in ER-treated WT and R cells (Figure 3A). Intriguingly, levels of NAD^+^ in untreated WT cells were significantly lower (p-value = 0.009) compared to R cells. NADP^+^ was significantly depleted in ER-treated WT cells (p-value = 0.03) but not ER-treated R cells (Figure 3B).

Furthermore, depletion of NAD+/NADH has been shown to leads to membrane depolarization, mitophagy and cell death via apoptosis or ferroptosis (McKay-Corkum et al., 2023; Zhu et al., 2022). Therefore, we examined effects of ER on cellular stability utilizing DiBAC4(3), a dye specific for studying plasma membrane polarization/depolarization in cells. Results presented in Figure 3C clearly shows that ER induced significant polarization of both cells within 4 h of treatment. Moreover, NCI/ADR-RES (R) cells were more sensitive to this depolarization than OVCAR-8 cells (WT).

### Erastin induces changes in energy metabolism

**FIGURE 4:**
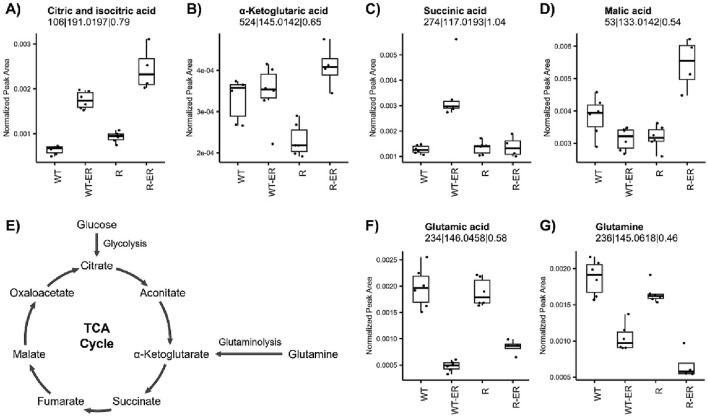
Influence of ER treatment on TCA cycle and glutaminolysis energy metabolism in WT and R cells. Metabolomics measurement of **(A)** iso- and citric acid, **(B)** α-ketoglutaric acid, **(C)** succinic acid, and **(D)** malic acid in the **(E)** TCA cycle. Glutaminolysis metabolites, **(F)** glutamic acid and **(G)** glutamine, measured by metabolomics.

Key energy metabolism pathways such as the tricarboxylic acid (TCA) cycle and glutaminolysis have been implicated in ferroptosis (Liu et al., 2023; Martinez-Reyes and Chandel, 2020). Untargeted metabolomics analysis indicated a significant increase in citric and isocitric acid in ER-treated WT and R cells (Figure 4A). Other TCA metabolites were differential following ER treatment in either WT or R cells (Figures 4B–D), such as α-ketoglutaric acid (p-value = 4.0 × 10^−4^) and malic acid (p-value = 1.7 × 10^−5^) which increased in ER-treated R cells. Conversely, succinic acid increased only in ER-treated WT cells (p-value = 7.3 × 10^−5^).

Glutamine serves as an important source of carbon to replenish the TCA cycle, as its degradation via glutaminolysis produces glutamate and subsequently the TCA cycle metabolite α-ketoglutarate (Figure 4E) as well as glutathione (Jin et al., 2023). Untargeted metabolomics analysis revealed that both glutamic acid and glutamine levels significantly decreased in WT and R cells in response to ER treatment (Figures 4F,G).

### Erastin induces altered lipid metabolism

**FIGURE 5:**
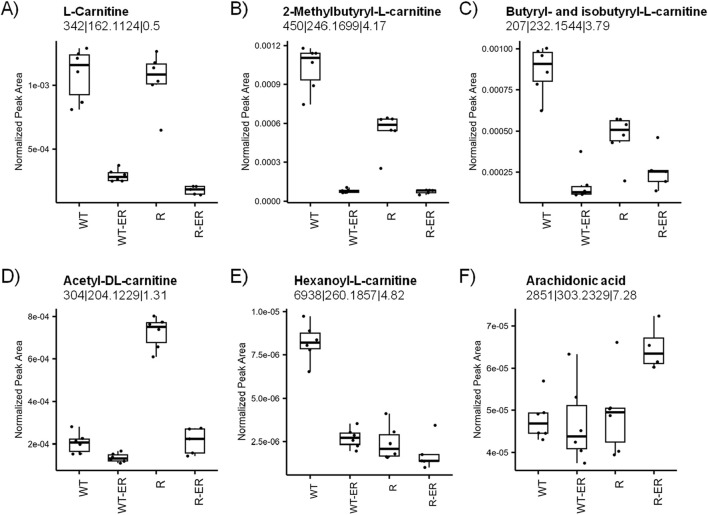
Modulation of lipid metabolism by ER in WT and R cells. Metabolomics measurement of **(A)** L-carnitine, **(B)** 2-methylbutyryl-L-carnitine, **(C)** iso- and butyryl-L-carnitine, **(D)** acetyl-DL-carnitine, **(E)** hexanoyl-L-carnitine, and **(F)** arachidonic acid.

Primary functions of L-carnitines are to transport activated long chain fatty acids (long chain fatty acyl-CoAs) into the mitochondria for degradation by β-oxidation and generation of ATP (Virmani and Cirulli, 2022; Farahzadi et al., 2023). As our previous studies in colon cancer cell lines had shown significant modulation of L-carnitines during drug-induced ferroptosis (Sinha et al., 2023), we sought to examine effects of ER on carnitines in ovarian cells. Metabolomics analysis indicated decreased levels of all identified L-carnitines (Figures 5A–E) in response to ER treatment. L-carnitine and two of its acyl analogues 2-methylbutyryl-L-carnitine and iso/butyryl-L-carnitine decreased in both WT and R cells, however this change was smaller for 2-methylbutyryl-L-carnitine and iso/butyryl-L-carnitine in R cells as the levels in untreated R cells were lower than untreated WT cells (p-values = 4.9 × 10^−6^ and 1.9 × 10^−4^, respectively). Acetyl-DL-carnitine levels were significantly higher in untreated R cells than WT cells (p-value = 5.8 × 10^−12^), whereas hexanoyl-L-carnitine was significantly higher in untreated WT cells than R (p-value = 6.6 × 10^−9^).

Ferroptosis results from the peroxidation of polyunsaturated fatty acids (PUFAs) in the cell membrane, such as arachidonic acid. PUFAs are continuously oxidized and reduced under normal cellular conditions; however, the depletion of GSH can lead to an accumulation of oxidized lipids, ultimately leading to cell membrane damage and cell death. Interestingly, arachidonic acid levels were similar in WT, WT-ER, and R cells, whereas R-ER was found to have a slight increase (p-value = 0.03, fold-change = 1.3, Figure 5F).

## Discussion

Our data show that OVCAR-8 (WT) and NCI/ADR-RES (R) cells treated with Erastin (ER) induced metabolomic differences resulting in, statistically significant changes in individual metabolites related to redox balance, energy, and lipid metabolism. Notably, we observed that WT and R cells had differential metabolomics profiles prior to ER treatment reflecting changes in metabolism. ER treatment induced large metabolomic changes as observed in the PCA plots (Figure 1) in positive and negative ionization mode, capturing metabolites converted to positive and negative ions, respectively. The change in overall profile implied substantial changes in metabolites, thus, we pursued univariate statistical analysis of the data focusing on major metabolomic processes.

As ER has been shown to cause GSH depletion by inhibiting Xc-transporter, leading to ROS/Lipid ROS formation in a wide variety of tumor cells (Zhao et al., 2020; Shintoku et al., 2017) loss of cellular GSH has become a hallmark of ferroptotic cell death and redox balance is central to the ferroptosis process. Untargeted metabolomics data revealed a significant reduction in GSH in WT-ER and R-ER cells compared to untreated WT and R cells. GSSG, a metabolite of glutathione oxidation, was decreased in WT-ER to a greater extent than in R-ER cells. The differences in initial GSH and GSSG levels between WT and R cells can make interpretation of the redox couple complicated, therefore, the ratio of GSH:GSSG was used (Figure 2C). Interestingly, the ratio of GSH:GSSG was similar between WT-ER and R-ER cells, indicating similar oxidative stress in both cell types. Soga et al. (Soga et al., 2006) have shown that decreases in GSH caused following treatment with acetaminophen induces increases in the formation of opthalmate (OPH) in liver. Formation of OPH in cells/blood has been proposed as a biomarker of oxidative stress (Soga et al., 2006; Dello et al., 2013). In this study, we also observed significant increases in OPH formation in both cell lines (Figure 2) with concomitant decreases in both GSH and taurine, a cellular antioxidant. Further exploration of the metabolomics data indicated a statistical difference in the levels of OPH (Figure 2D). An increase in OPH was observed in the ER-treated cells with the greatest levels observed in R-ER cells. OPH is a reported biomarker of oxidative stress in cells/blood (Soga et al., 2006; Dello et al., 2013); however, the precise roles of OPH are unknown. It is proposed that OPH serves as a tripeptide regulator of glutathione (Schomakers et al., 2024). We have previously reported decreases in taurine during NCX4040-induced ferroptosis in colon cancer cells (Sinha et al., 2023). Therefore, our study lends support for ophthalmate formation in cells along with taurine as possible biomarkers for ferroptotic cell death.

Our studies also showed that nicotinamide adenine dinucleotides, NAD+/NADP+ were decreased by ER treatment in both OVCAR-8 (WT) and NCI/ADR-RES (R) cells (Figure 5). However, a significantly more decrease was observed in the OVCAR-8 cells than in the resistant variant. NAD+ is an essential cofactor that supports enzymes involved in a number of cellular processes, including metabolic functions, such as glycolysis, oxidative phosphorylation, maintenance of redox balance, TCA cycle and ATP production (Biniecka et al., 2023; McKay-Corkum et al., 2023; Massudi et al., 2012; Wu et al., 2016). Furthermore, it serves as a substrate for several NAD-degrading enzymes, including poly ADP-ribose polymerases (PARP). Several recent studies have shown that depletion of cellular NAD+/NADH leads to cell death through mitochondrial depolarization (McKay-Corkum et al., 2023; Sun et al., 2023). Although mitochondrial depolarization was not directly investigated in our studies, our findings indicate that ER significantly induced plasma membrane depolarization in both cell types, with a more pronounced effect observed in NCI/ADR-RES cells. It appears that extensive oxidation of polyunsaturated fatty acid (PUFA)-containing phospholipids might modify membrane structure and increase membrane permeability, eventually resulting in plasma membrane rupture in response to accumulation of lipid-ROS, and cell death.

Exploration of the TCA cycle metabolites detected in our untargeted metabolomics experiment indicated substantial changes in TCA organic acid, uniquely changing between WT-ER and R-ER cells (Figure 4). Citric acid was increased in ER-treated cells with slightly greater levels observed in R-ER, which may reflect a greater level of glycolysis in as citrate is the catabolite of acetyl-CoA, which in turn is produced from pyruvate. Glycolysis was poorly measured via our untargeted assay and, therefore, we cannot conclusively determine the rationale for the observed increase. Other notable changes in TCA metabolites were the specific increase in succinic acid observed in WT-ER cells and the explicit increase in malic acid observed in R-ER cells. This would imply differences in metabolism or flux through the TCA cycle.

Erastin also modulated glutamine and glutamic acid in WT and R cells, slightly more in the resistant variants. Glutamine and glutamic acid are utilized both in normal and cancer cell growth, however, cancer cells require more biofuel than normal tissues for energy supply, anti-oxidation activity and biomass production (Yi et al., 2019). Glutamate is a source of carbon to replenish the tricarboxylic acid (TCA) cycle, is a precursor for glutathione, and serves as a precursor to nucleotides and lipid synthesis via reductive carboxylation. Furthermore, glutamine can contribute to the intracellular acetyl-CoA pool as WT cell as to NAD+ and NADH levels, suggesting a potential role in regulating histone and protein acetylation levels and patterns (Jin et al., 2023). Decreases in glutamate have significant consequences for generating ATP, fatty acids, purines and pyrimidines. Furthermore, the observed decrease in glutamic acid levels in ER-treated cells would negatively influence the levels of GSH since biosynthesis of GSH is dependent on glutamic acid. Thus, modulation of glutamic acid by ER-treatment, causes a substantial metabolomic challenge in balancing energy production via glutaminolysis and glutamic acid availability for redox balance. Therefore, this may serve as an important balance between competing pathways in ferroptosis.

Our studies also suggest that lipid metabolism induced by ferroptosis inducers may play significant role in cell death (Figure 5). In WT and R ovarian cells, ER significantly decreased multiple acylcarnitines in our metabolomics data. Acylcarnitines have been shown to play an important role in the transport of fatty acids for beta-oxidation for energy production in cells. Thus, decreases in Acylcarnitines induced by ER would result in decreased ATP production, leading to cell death (Farahzadi et al., 2023). Furthermore, acylcarnitines protects cells from oxidative damage by protecting detoxifying enzymes (SOD, Catalase and GSH-dependent peroxidases) (Gomez-Amores et al., 2006). Thus, decreases in carnitines by ER and ferroptosis inducers have important consequences for energy production and cell survival by protecting the mitochondrial membrane integrity against ROS attack, decreasing lipid peroxidation and ferroptosis. We believe that carnitines represent an important class of compounds in ferroptosis and should therefore be considered key biomarkers for this form of cell death. While in this study we have only focused on two ovarian tumors cells to study Erastin-induced metabolic changes, our conclusions need to be confirmed in other ovarian tumor cells as well as in patients-derived ovarian tumor samples for a broader applicability of our findings.

**FIGURE 6:**
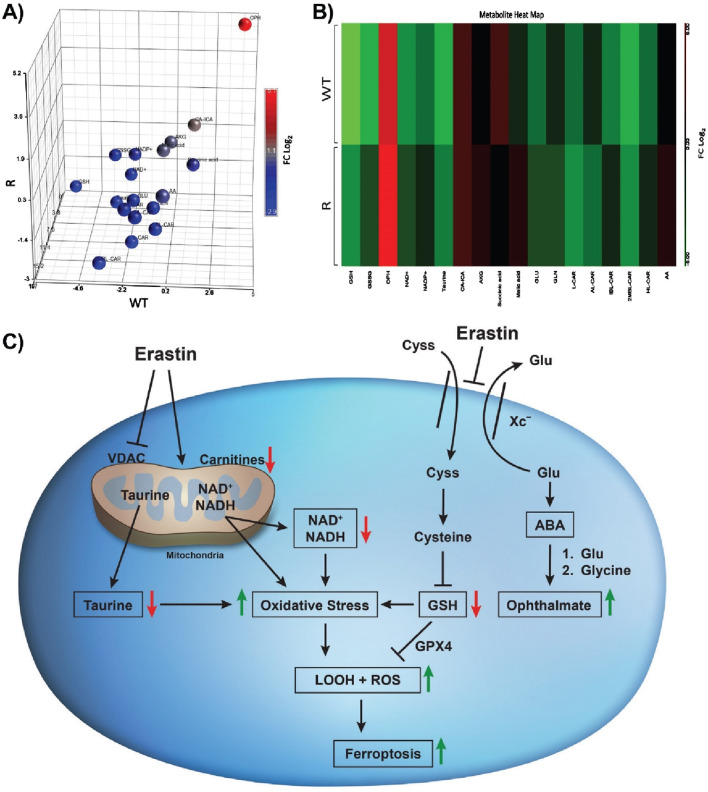
Principal component analysis **(A)** heat map **(B)** generated from the relative intensity of biomarkers formed along with Erastin-induced changes in cellular pathways **(C)** leading to oxidative stress and cell death in OVCAR-8 and NCI/ADR-RES ovarian tumor cells following 24 h of drug treatment. OPH is formed after the conversion of glutamate to 2-aminobutyric acid (ABA), which is then conjugated with glutamine by glutamine-cysteine ligase. This is followed by the attachment of glycine, a process catalyzed by glutathione synthetase.

Principal component analysis (PCA), the heat map of biomarkers (Supplementary Table S1) induced by ER treatment of OVCAR-8 and NCI/ADR-RES tumor cells and cellular pathways leading to ferroptosis induced by ER are summarized in Figure 6.

## Conclusion

In conclusion, untargeted metabolomics studies in human ovarian tumor, OVCAR-8 and NCI/ADR-RES cells, identified a number of important metabolites following treatment with ER. These studies have identified a number of pathways that may be important for the initiation of cell death by ferroptosis inducers. We found significant depletion of cellular GSH in both cell types, a first step in ROS and Lipid ROS formation for the initiation of ferroptotic cell death. This loss of GSH also resulted in increases in ophthalmate formation and concomitant decreases in taurine in both cells, indicating significant oxidative stress in both cells. These studies also showed that ER treatment significantly decreased carnitines which are essential for energy production and mitochondrial protection against lipid peroxidation and detoxifying enzymes. Additionally, we found that ER treatment also significantly decreased glutamine/glutamate in these cells. This would further support that ER increased oxidative stress, and decreased energy production in these cells. These studies suggest that depletion of GSH, increased formation of ophthalmate, decreases in cellular taurine, carnitines and glutamate strongly point towards potential biomarkers of ferroptosis in tumor cells.

## Data Availability

The data presented in the study are deposited in the MassIVE repository, accession number MSV000096771.

## Abbreviations

ER, Erastin; VADC, voltage-dependent anion channel, Xc-, glutamate/cystine antiporter, ROS, Reactive Oxygen Species; GPX4, glutathione peroxidase 4; MDR1, multi-drug resistance1; OVCAR-8, (W); NCI/ADR-RES, (R).

## References

[B1] Baliou S. Adamaki M. Ioannou P. Pappa A. Panayiotidis M. I. Spandidos D. A. (2021). Protective role of taurine against oxidative stress (Review). Mol. Med. Rep. 24, 605. 10.3892/mmr.2021.12242 34184084 PMC8240184

[B2] Batist G. Tulpule A. Sinha B. K. Katki A. G. Myers C. E. Cowan K. H. (1986). Overexpression of a novel anionic glutathione transferase in multidrug-resistant human breast cancer cells. J. Biol. Chem. 261, 15544–15549. 10.1016/s0021-9258(18)66748-1 3782078

[B3] Biniecka P. Matsumoto S. Belotti A. Joussot J. Bai J. F. Majjigapu S. R. (2023). Anticancer activities of novel nicotinamide phosphoribosyltransferase inhibitors in hematological malignancies. Molecules 28, 1897. 10.3390/molecules28041897 36838885 PMC9967653

[B4] Chen T. H. Wang H. C. Chang C. J. Lee S. Y. (2024). Mitochondrial glutathione in cellular redox homeostasis and disease manifestation. Int. J. Mol. Sci. 25, 1314. 10.3390/ijms25021314 38279310 PMC10816320

[B5] Chen X. Comish P. B. Tang D. Kang R. (2021). Characteristics and biomarkers of ferroptosis. Front. Cell Dev. Biol. 9, 637162. 10.3389/fcell.2021.637162 33553189 PMC7859349

[B6] Cheng Q. Bao L. Li M. Chang K. Yi X. (2021). Erastin synergizes with cisplatin via ferroptosis to inhibit ovarian cancer growth *in vitro* and *in vivo* . J. Obstet. Gynaecol. Res. 47, 2481–2491. 10.1111/jog.14779 33882617

[B7] Cortez A. J. Tudrej P. Kujawa K. A. Lisowska K. M. (2018). Advances in ovarian cancer therapy. Cancer Chemother. Pharmacol. 81, 17–38. 10.1007/s00280-017-3501-8 29249039 PMC5754410

[B8] Cowan K. H. Batist G. Tulpule A. Sinha B. K. Myers C. E. (1986). Similar biochemical changes associated with multidrug resistance in human breast cancer cells and carcinogen-induced resistance to xenobiotics in rats. Proc. Natl. Acad. Sci. U. S. A. 83, 9328–9332. 10.1073/pnas.83.24.9328 3540935 PMC387131

[B9] DeHart D. N. Fang D. Heslop K. Li L. Lemasters J. J. Maldonado E. N. (2018). Opening of voltage dependent anion channels promotes reactive oxygen species generation, mitochondrial dysfunction and cell death in cancer cells. Biochem. Pharmacol. 148, 155–162. 10.1016/j.bcp.2017.12.022 29289511 PMC5909406

[B10] Dello S. A. Neis E. P. de Jong M. C. van Eijk H. M. Kicken C. H. Olde Damink S. W. (2013). Systematic review of ophthalmate as a novel biomarker of hepatic glutathione depletion. Clin. Nutr. 32, 325–330. 10.1016/j.clnu.2012.10.008 23182341

[B11] Dixon S. J. Lemberg K. M. Lamprecht M. R. Skouta R. Zaitsev E. M. Gleason C. E. (2012). Ferroptosis: an iron-dependent form of nonapoptotic cell death. Cell 149, 1060–1072. 10.1016/j.cell.2012.03.042 22632970 PMC3367386

[B12] Dixon S. J. Patel D. N. Welsch M. Skouta R. Lee E. D. Hayano M. (2014). Pharmacological inhibition of cystine-glutamate exchange induces endoplasmic reticulum stress and ferroptosis. Elife 3, e02523. 10.7554/eLife.02523 24844246 PMC4054777

[B13] Farahzadi R. Hejazi M. S. Molavi O. Pishgahzadeh E. Montazersaheb S. Jafari S. (2023). Clinical significance of carnitine in the treatment of cancer: from traffic to the regulation. Oxid. Med. Cell Longev. 2023, 9328344. 10.1155/2023/9328344 37600065 PMC10435298

[B14] Fiehn O. Robertson D. Griffin J. van der Werf M. Nikolau B. Morrison N. (2007). The metabolomics standards initiative (MSI). Metabolomics 3, 175–178. 10.1007/s11306-007-0070-6

[B15] Galaris D. Barbouti A. Pantopoulos K. (2019). Iron homeostasis and oxidative stress: an intimate relationship. Biochim. Biophys. Acta Mol. Cell Res. 1866, 118535. 10.1016/j.bbamcr.2019.118535 31446062

[B16] Gomez-Amores L. Mate A. Revilla E. Santa-Maria C. Vazquez C. M. (2006). Antioxidant activity of propionyl-L-carnitine in liver and heart of spontaneously hypertensive rats. Life Sci. 78, 1945–1952. 10.1016/j.lfs.2005.08.023 16263137

[B17] Jiang L. Kon N. Li T. Wang S. J. Su T. Hibshoosh H. (2015). Ferroptosis as a p53-mediated activity during tumour suppression. Nature 520, 57–62. 10.1038/nature14344 25799988 PMC4455927

[B18] Jin J. Byun J. K. Choi Y. K. Park K. G. (2023). Targeting glutamine metabolism as a therapeutic strategy for cancer. Exp. Mol. Med. 55, 706–715. 10.1038/s12276-023-00971-9 37009798 PMC10167356

[B19] Kang R. Kroemer G. Tang D. (2019). The tumor suppressor protein p53 and the ferroptosis network. Free Radic. Biol. Med. 133, 162–168. 10.1016/j.freeradbiomed.2018.05.074 29800655 PMC6251771

[B20] Kemp M. Go Y. M. Jones D. P. (2008). Nonequilibrium thermodynamics of thiol/disulfide redox systems: a perspective on redox systems biology. Free Radic. Biol. Med. 44, 921–937. 10.1016/j.freeradbiomed.2007.11.008 18155672 PMC2587159

[B21] Kuang Y. Yang K. Meng L. Mao Y. Xu F. Liu H. (2023). Identification and validation of ferroptosis-related biomarkers and the related pathogenesis in precancerous lesions of gastric cancer. Sci. Rep. 13, 16074. 10.1038/s41598-023-43198-4 37752199 PMC10522668

[B22] Liu Y. Lu S. Wu L. L. Yang L. Yang L. Wang J. (2023). The diversified role of mitochondria in ferroptosis in cancer. Cell Death Dis. 14, 519. 10.1038/s41419-023-06045-y 37580393 PMC10425449

[B23] Maldonado E. N. Sheldon K. L. DeHart D. N. Patnaik J. Manevich Y. Townsend D. M. (2013). Voltage-dependent anion channels modulate mitochondrial metabolism in cancer cells: regulation by free tubulin and erastin. J. Biol. Chem. 288, 11920–11929. 10.1074/jbc.M112.433847 23471966 PMC3636879

[B24] Martinez-Reyes I. Chandel N. S. (2020). Mitochondrial TCA cycle metabolites control physiology and disease. Nat. Commun. 11, 102. 10.1038/s41467-019-13668-3 31900386 PMC6941980

[B25] Massudi H. Grant R. Guillemin G. J. Braidy N. (2012). NAD+ metabolism and oxidative stress: the golden nucleotide on a crown of thorns. Redox Rep. 17, 28–46. 10.1179/1351000212Y.0000000001 22340513 PMC6837626

[B26] McKay-Corkum G. B. Collins V. J. Yeung C. Ito T. Issaq S. H. Holland D. (2023). Inhibition of NAD+-Dependent metabolic processes induces cellular necrosis and tumor regression in rhabdomyosarcoma models. Clin. Cancer Res. 29, 4479–4491. 10.1158/1078-0432.CCR-23-0200 37616468 PMC10841338

[B27] Mendez Hernandez R. Ramasco Rueda F. (2023). Biomarkers as prognostic predictors and therapeutic guide in critically ill patients: clinical evidence. J. Pers. Med. 13, 333. 10.3390/jpm13020333 36836567 PMC9965041

[B28] Overdahl K. E. Collier J. B. Jetten A. M. Jarmusch A. K. (2023). Signal response evaluation applied to untargeted mass spectrometry data to improve data interpretability. J. Am. Soc. Mass Spectrom. 34, 1941–1948. 10.1021/jasms.3c00220 37524076 PMC10485927

[B29] Schomakers B. V. Jillings S. L. van Weeghel M. Vaz F. M. Salomons G. S. Janssens G. E. (2024). Ophthalmic acid is a glutathione regulating tripeptide. FEBS J. 291, 3317–3330. 10.1111/febs.17061 38245827

[B30] Shintoku R. Takigawa Y. Yamada K. Kubota C. Yoshimoto Y. Takeuchi T. (2017). Lipoxygenase-mediated generation of lipid peroxides enhances ferroptosis induced by erastin and RSL3. Cancer Sci. 108, 2187–2194. 10.1111/cas.13380 28837253 PMC5666033

[B31] Sinha B. K. Bortner C. D. Jarmusch A. K. Tokar E. J. Murphy C. Wu X. (2023). Ferroptosis-mediated cell death induced by NCX4040, the non-steroidal nitric oxide donor, in human colorectal cancer cells: implications in therapy. Cells 12, 1626. 10.3390/cells12121626 37371096 PMC10297642

[B32] Sinha B. K. Murphy C. Brown S. M. Silver B. B. Tokar E. J. Bortner C. D. (2024). Mechanisms of cell death induced by erastin in human ovarian tumor cells. Int. J. Mol. Sci. 25, 8666. 10.3390/ijms25168666 39201357 PMC11355013

[B33] Soga T. Baran R. Suematsu M. Ueno Y. Ikeda S. Sakurakawa T. (2006). Differential metabolomics reveals ophthalmic acid as an oxidative stress biomarker indicating hepatic glutathione consumption. J. Biol. Chem. 281, 16768–16776. 10.1074/jbc.M601876200 16608839

[B34] Sui X. Zhang R. Liu S. Duan T. Zhai L. Zhang M. (2018). RSL3 drives ferroptosis through GPX4 inactivation and ROS production in colorectal cancer. Front. Pharmacol. 9, 1371. 10.3389/fphar.2018.01371 30524291 PMC6262051

[B35] Sun C. Seranova E. Cohen M. A. Chipara M. Roberts J. Astuti D. (2023). NAD depletion mediates cytotoxicity in human neurons with autophagy deficiency. Cell Rep. 42, 112372. 10.1016/j.celrep.2023.112372 37086404 PMC10556436

[B36] Surai P. F. Earle-Payne K. Kidd M. T. (2021). Taurine as a natural antioxidant: from direct antioxidant effects to protective action in various toxicological models. Antioxidants (Basel) 10, 1876. 10.3390/antiox10121876 34942978 PMC8698923

[B37] Virmani M. A. Cirulli M. (2022). The role of l-carnitine in mitochondria, prevention of metabolic inflexibility and disease initiation. Int. J. Mol. Sci. 23, 2717. 10.3390/ijms23052717 35269860 PMC8910660

[B38] Wang L. Wang X. Zhu X. Zhong L. Jiang Q. Wang Y. (2024). Drug resistance in ovarian cancer: from mechanism to clinical trial. Mol. Cancer 23, 66. 10.1186/s12943-024-01967-3 38539161 PMC10976737

[B39] Wu J. Jin Z. Zheng H. Yan L. J. (2016). Sources and implications of NADH/NAD(+) redox imbalance in diabetes and its complications. Diabetes Metab. Syndr. Obes. 9, 145–153. 10.2147/DMSO.S106087 27274295 PMC4869616

[B40] Yagoda N. von Rechenberg M. Zaganjor E. Bauer A. J. Yang W. S. Fridman D. J. (2007). RAS-RAF-MEK-dependent oxidative cell death involving voltage-dependent anion channels. Nature 447, 864–868. 10.1038/nature05859 17568748 PMC3047570

[B41] Yang W. S. Stockwell B. R. (2016). Ferroptosis: death by lipid peroxidation. Trends Cell Biol. 26, 165–176. 10.1016/j.tcb.2015.10.014 26653790 PMC4764384

[B42] Yi H. Talmon G. Wang J. (2019). Glutamate in cancers: from metabolism to signaling. J. Biomed. Res. 34, 260–270. 10.7555/JBR.34.20190037 32594024 PMC7386414

[B43] Yu Y. Xie Y. Cao L. Yang L. Yang M. Lotze M. T. (2015). The ferroptosis inducer erastin enhances sensitivity of acute myeloid leukemia cells to chemotherapeutic agents. Mol. Cell Oncol. 2, e1054549. 10.1080/23723556.2015.1054549 27308510 PMC4905356

[B44] Zhao Y. Li Y. Zhang R. Wang F. Wang T. Jiao Y. (2020). The role of erastin in ferroptosis and its prospects in cancer therapy. Onco Targets Ther. 13, 5429–5441. 10.2147/OTT.S254995 32606760 PMC7295539

[B45] Zhu J. Gillissen B. Dang Tran D. L. May S. Ulrich C. Stockfleth E. (2022). Inhibition of cell proliferation and cell viability by sinecatechins in cutaneous SCC cells is related to an imbalance of ROS and loss of mitochondrial membrane potential. Antioxidants (Basel) 11, 1416. 10.3390/antiox11071416 35883905 PMC9312260

