# Supplementary material for: Metabolic consequences of erastin-induced ferroptosis in human ovarian cancer cells: an untargeted metabolomics study
Source: Front Mol Biosci. 2025 Jan 20;11:1520876. doi: 10.3389/fmolb.2024.1520876 (PMC11788483; doi:10.3389/fmolb.2024.1520876)
Supplement: Supplementary file 1 [file Table1.docx]

**Table1-S1.** Tabulated fold changes and adjusted p-values for metabolites of interest

| Metabolite | Comparison | | Log_2_ Fold Change | Adjusted p-value |
| --- | --- | --- | --- | --- |
| Reduced glutathione (GSH) | WT Control:ER treated | -6.2 | | 1.8 x 10^-12^ |
|  | R Control:ER treated | -2.6 | | 5.3 x 10^-6^ |
| Oxidized glutathione (GSSG) | WT Control:ER treated | -3.6 | | 4.6 x 10^-7^ |
|  | R Control:ER treated | -0.9 | | 0.060 |
| Opthalamic acid (OPH) | WT Control:ER treated | 4.2 | | 4.3 x 10^-8^ |
|  | R Control:ER treated | 5.1 | | 1.0 x 10^-10^ |
| Nicotinamide adenine dinucleotide (NAD+) | WT Control:ER treated | -2.5 | | 1.2 x 10-4 |
|  | R Control:ER treated | -1.4 | | 3.5 x 10-5 |
| Nicotinamide adenine dinucleotide phosphate (NADP+) | WT Control:ER treated | -2.2 | | 0.030 |
|  | R Control:ER treated | -0.3 | | 0.89 |
| Taurine | WT Control:ER treated | -3.3 | | 4.2 x 10^-12^ |
|  | R Control:ER treated | -2.3 | | 2.4 x 10^-7^ |
| Citric/isocitric acid | WT Control:ER treated | 1.4 | | 1.2 x 10^-6^ |
|  | R Control:ER treated | 1.4 | | 6.4 x 10^-8^ |
| α-Ketoglutaric acid | WT Control:ER treated | 0.06 | | 0.97 |
|  | R Control:ER treated | 0.8 | | 4.0 x 10^-4^ |
| Succinic acid | WT Control:ER treated | 1.4 | | 7.3 x 10^-5^ |
|  | R Control:ER treated | 0.04 | | 1.0 |
| Malic acid | WT Control:ER treated | -0.3 | | 0.14 |
|  | R Control:ER treated | 0.8 | | 1.7 x 10^-5^ |
| Glutamic acid | WT Control:ER treated | -2.0 | | 5.0 x 10^-8^ |
|  | R Control:ER treated | -1.2 | | 4.0 x 10^-5^ |
| Glutamine | WT Control:ER treated | 0.8 | | 4.4 x 10^-6^ |
|  | R Control:ER treated | -1.3 | | 1.9 x 10^-6^ |
| L-carnitine | WT Control:ER treated | -2.0 | | 2.0 x 10^-7^ |
|  | R Control:ER treated | -2.6 | | 1.1 x 10^-7^ |
| Acetyl-DL-carnitine | WT Control:ER treated | -0.6 | | 0.17 |
|  | R Control:ER treated | -1.8 | | 1.9 x 10^-11^ |
| Butyrl/isobutyrl-L-carnitine | WT Control:ER treated | -2.3 | | 1.0 x 10^-7^ |
|  | R Control:ER treated | 0.8 | | 0.08 |
| 2-Methylbutyryl-L-carnitine | WT Control:ER treated | -3.7 | | 1.1 x 10^-10^ |
|  | R Control:ER treated | -2.9 | | 1.4 x 10^-5^ |
| Hexanoyl-L-carnitine | WT Control:ER treated | -1.6 | | 1.5 x 10^-8^ |
|  | R Control:ER treated | -0.7 | | 0.040 |
| Arachidonic acid | WT Control:ER treated | -0.03 | | 1.0 |
|  | R Control:ER treated | 0.40 | | 0.03 |
