# Supplementary material for: Metabolic consequences of erastin-induced ferroptosis in human ovarian cancer cells: an untargeted metabolomics study
Source: Front Mol Biosci. 2025 Jan 20;11:1520876. doi: 10.3389/fmolb.2024.1520876 (PMC11788483; doi:10.3389/fmolb.2024.1520876)
Supplement: Supplementary file 2 [file Image1.pdf]

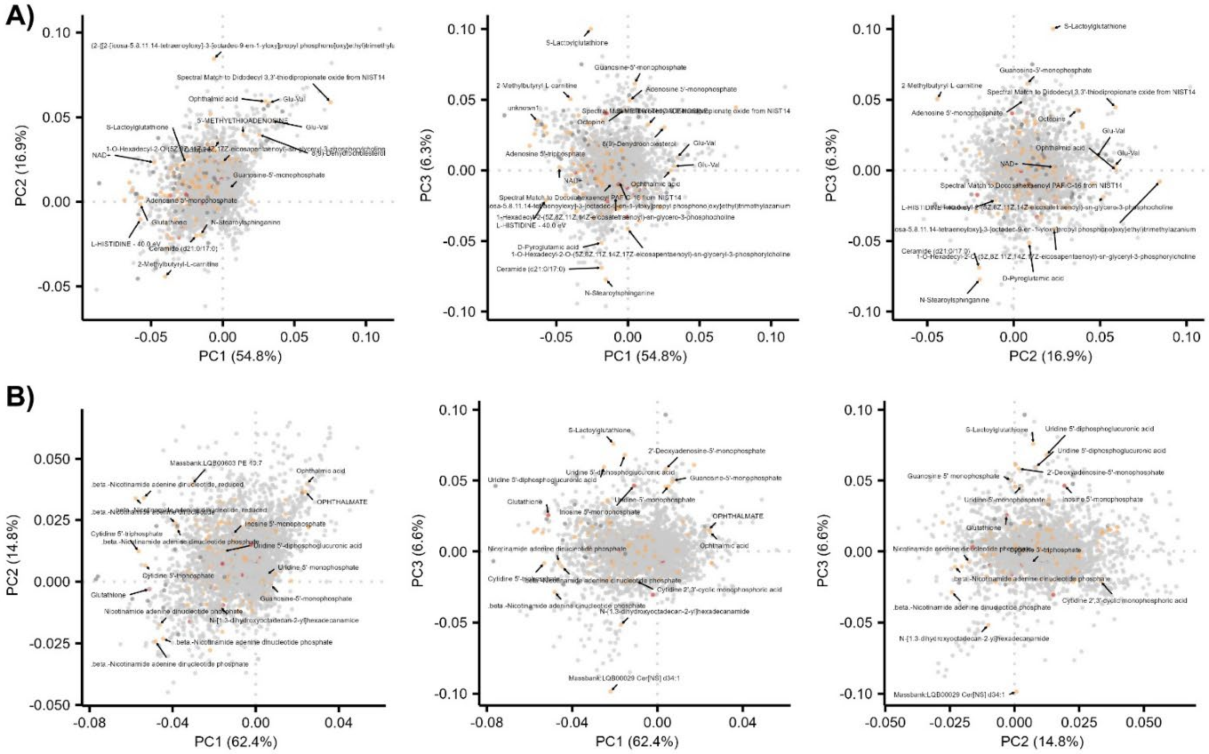

Supporting Information

Supplementary Figure 1: **:** PCA of WT and R cells prior to and 24 h after Erastin treatment. PCA score plots for principal components 1 through 3: (**A**) positive ionization mode data and (**B**) negative ionization mode data.
